# Experimental annotation of the human pathogen *Candida albicans *coding and noncoding transcribed regions using high-resolution tiling arrays

**DOI:** 10.1186/gb-2010-11-7-r71

**Published:** 2010-07-09

**Authors:** Adnane Sellam, Hervé Hogues, Christopher Askew, Faiza Tebbji, Marco van het Hoog, Hugo Lavoie, Carol A Kumamoto, Malcolm Whiteway, André Nantel

**Affiliations:** 1 Biotechnology Research Institute, National Research Council of Canada, 6100 Royalmount, Montréal, Québec, H4P 2R2, Canada; 2 Department of Anatomy and Cell Biology, McGill University, 3640 University Street, Montréal, Québec, H3A 1B1, Canada; 3 Department of Biology, McGill University, 1205 Docteur Penfield, Montréal, Québec, H3A 1B1, Canada; 4 Intracellular Signaling Laboratory, Institute of Research in Immunology and Cancer (IRIC), University of Montreal, 2900 boulevard Édouard-Montpetit, Montreal, Quebec, H3C 3J7, Canada; 5 Department of Molecular Biology and Microbiology, Tufts University, 136 Harrison Avenue, Boston, MA 02111, USA

## Abstract

**Background:**

Compared to other model organisms and despite the clinical relevance of the pathogenic yeast *Candida albicans*, no comprehensive analysis has been done to provide experimental support of its *in silico*-based genome annotation.

**Results:**

We have undertaken a genome-wide experimental annotation to accurately uncover the transcriptional landscape of the pathogenic yeast *C. albicans *using strand-specific high-density tiling arrays. RNAs were purified from cells growing under conditions relevant to *C. albicans *pathogenicity, including biofilm, lab-grown yeast and serum-induced hyphae, as well as cells isolated from the mouse caecum. This work provides a genome-wide experimental validation for a large number of predicted ORFs for which transcription had not been detected by other approaches. Additionally, we identified more than 2,000 novel transcriptional segments, including new ORFs and exons, non-coding RNAs (ncRNAs) as well as convincing cases of antisense gene transcription. We also characterized the 5' and 3' UTRs of expressed ORFs, and established that genes with long 5' UTRs are significantly enriched in regulatory functions controlling filamentous growth. Furthermore, we found that genomic regions adjacent to telomeres harbor a cluster of expressed ncRNAs. To validate and confirm new ncRNA candidates, we adapted an iterative strategy combining both genome-wide occupancy of the different subunits of RNA polymerases I, II and III and expression data. This comprehensive approach allowed the identification of different families of ncRNAs.

**Conclusions:**

In summary, we provide a comprehensive expression atlas that covers relevant *C. albicans *pathogenic developmental stages in addition to the discovery of new ORF and non-coding genetic elements.

## Background

*Candida albicans *is an opportunistic pathogen responsible for various non life-threatening infections, such as oral thrush and vaginitis, and accounts for more than half of all *Candida *infections [1,2]. This pathogen is also a major cause of morbidity and mortality in bloodstream infections, especially in immunosuppressed individuals. *C. albicans *can also colonize various biomaterials, such as urinary and vascular catheters, and ventricular assist devices, and readily forms dense biofilms that are resistant to most antifungal drugs [3]. The ability of this fungus to switch from yeast to filamentous forms (true hyphae or pseudohyphae) is also a crucial determinant for host invasion and thus virulence [4]. Because of the challenges of drug resistance [5-7] and the eukaryotic nature of *C. albicans*, which makes it similar to its human host, extensive efforts are being made to identify specific new drug targets for therapeutic intervention.

The *C. albicans *genome has been the subject of many curated annotations that have resulted in the current comprehensive physical genomic map [8-11]. Recently, the genome sequences of six further species from the *Candida *clade have been released. Comparative analysis of these genomes revealed a significant expansion of gene families associated with virulence compared to non-pathogenic yeasts [12]. In addition, this work uncovered an unexpected divergence in the mechanisms controlling mating and meiosis in this clade. Given the high conservation of protein-coding sequence within the six *Candida *species, Butler *et al*. [12] undertook a comparative annotation to revise the genome sequence of *C. albicans *and identified 91 new or updated ORFs.

Genome sequencing followed by *in silico*-based annotation is the critical first step required to gain a comprehensive insight into the genetic features underlying different aspects of an organism's biology. To establish a more comprehensive and accurate layout of these features, *in silico *methods must be complemented by transcriptome or proteome investigations. Recent advances taking advantage of the high-throughput potential of whole-genome tiling microarrays or cDNA sequencing contributed significantly to the discovery of novel sites of active transcription missed by computational gene prediction (reviewed in [13-15]). Tiling array technology has revealed several unexpected hidden features of the eukaryotic transcriptome, including antisense (AS) transcription, non-coding RNAs (ncRNAs) as well as complex transcriptional architectures such as nested genes [16-22]. The use of tiling arrays has also been useful for mapping a variety of epigenetic marks in eukaryotes and uncovering the complex network of mechanisms involved in transcriptional regulation associated with chromatin dynamics [23-25]. Here we have undertaken a genome-wide experimental annotation using a strand-specific high-density tiling array that allows us to accurately uncover the transcriptional landscape of *C. albicans*. The main purposes of this work were: the experimental validation of computational-based genome annotations in *C. albicans*; the discovery of new coding and non-coding genetic elements for future studies; the identification of new functional features associated with the transcriptome organization; and the annotation of class I, II and III genes using an unbiased methodology that combines data from the genome-wide occupancy of different subunits of RNA polymerases (RNAPs) I, II and III with data from transcriptome studies.

## Results and discussion

To illuminate the transcriptional landscape of the pathogenic fungus *C. albicans*, we tiled both Watson and Crick strands of the whole genome with 240,798 60-mer probes each overlapping by 1 bp. Total RNA was purified from cells growing under various conditions relevant to *C. albicans *pathogenicity; specifically growing as a biofilm, as hyphae and as a commensal within the mouse caecum. RNA from cells growing as yeast in YPD at 30°C were used as a reference for each condition.

### Transcript mapping reveals extensive transcription in *C. albicans*

For each condition, thresholds were determined empirically based on the 95th percentile of signal intensities of non-conserved intergenic regions as described in the Materials and methods section. After combining expression data for all the tested conditions, transcription activity was detected for 72% of the 6,193 nuclear genes, including 4,402 ORFs, 4 pseudogenes, 67 tRNAs, 108 retrotransposons and 7 ncRNAs (5 small nuclear RNAs (snRNAs), 1 small nucleolar RNA (snoRNA) and the rRNA) (Table 1). The remaining 28% of the genomic features not detected in this study could be due to the fact that they are not used in our conditions, and an analysis of Gene Ontology (GO) functional categories of these unexpressed genes revealed a significant enrichment in functions related to the accomplishment of the parasexual cycle in *C. albicans*, including ascospore wall assembly (*P *= 1.74e-05), meiosis (*P *= 1.33e-02) and synapsis (*P *= 8.64e-04) (Additional file 1).

**Table 1:** Number of *Candida *Genome Database-annotated features whose expression was detected in the current study

Features	CGD	This study	Coverage
ORF	6,197	4,402	71%
Retrotransposon and LTR	129	108	83%
Pseudogene	8	5	62.5%
tRNA	156	67	43%
snRNA	5	5	100%
snoRNA	1	1	100%

CGD, *Candida *Genome Database; LTR, long terminal repeat.

A large number of transcribed segments, or transfrags [26], were detected in intergenic regions devoid of existing annotation. Transfrags were identified on the basis of two or more consecutive probes exhibiting intensities above the threshold, together with separation by at least 120 bp from any currently annotated elements. Using these criteria, a total of 2,172 transfrags were detected and mapped (Additional file 2). Interestingly, 31% of the intergenic transcribed units (680 transfrags) display significant sequence conservation (*e*-value < 10^-10^) with *Candida dubliniensis*, suggesting the existence of functional genetic elements.

### Features of transcribed regions in the *C. albicans *genome

**Figure 1:**
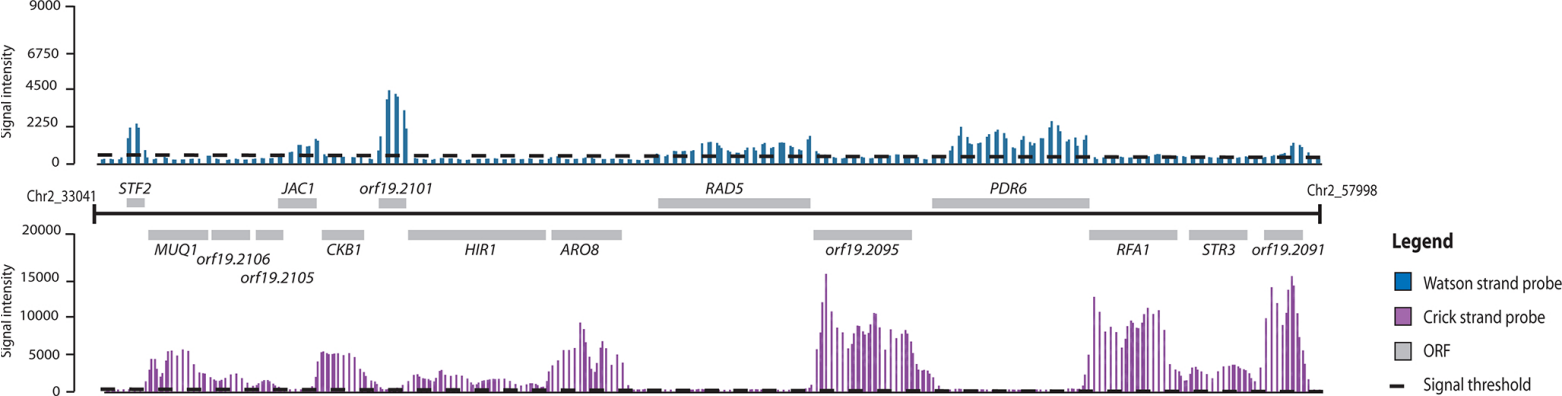
**Genome-wide view of a sample region of *C. albicans *chromosome 2**. Hybridization intensities for probes are provided as vertical bars along Watson (blue) and Crick (red) strands. The cutoff for signal probes is indicated with a dashed line corresponding to a fluorescence intensity of 777 and 655 for Watson and Crick strands, respectively. Annotated ORFs are depicted as grey boxes aligned to their own chromosomal coordinates.

**Figure 2:**
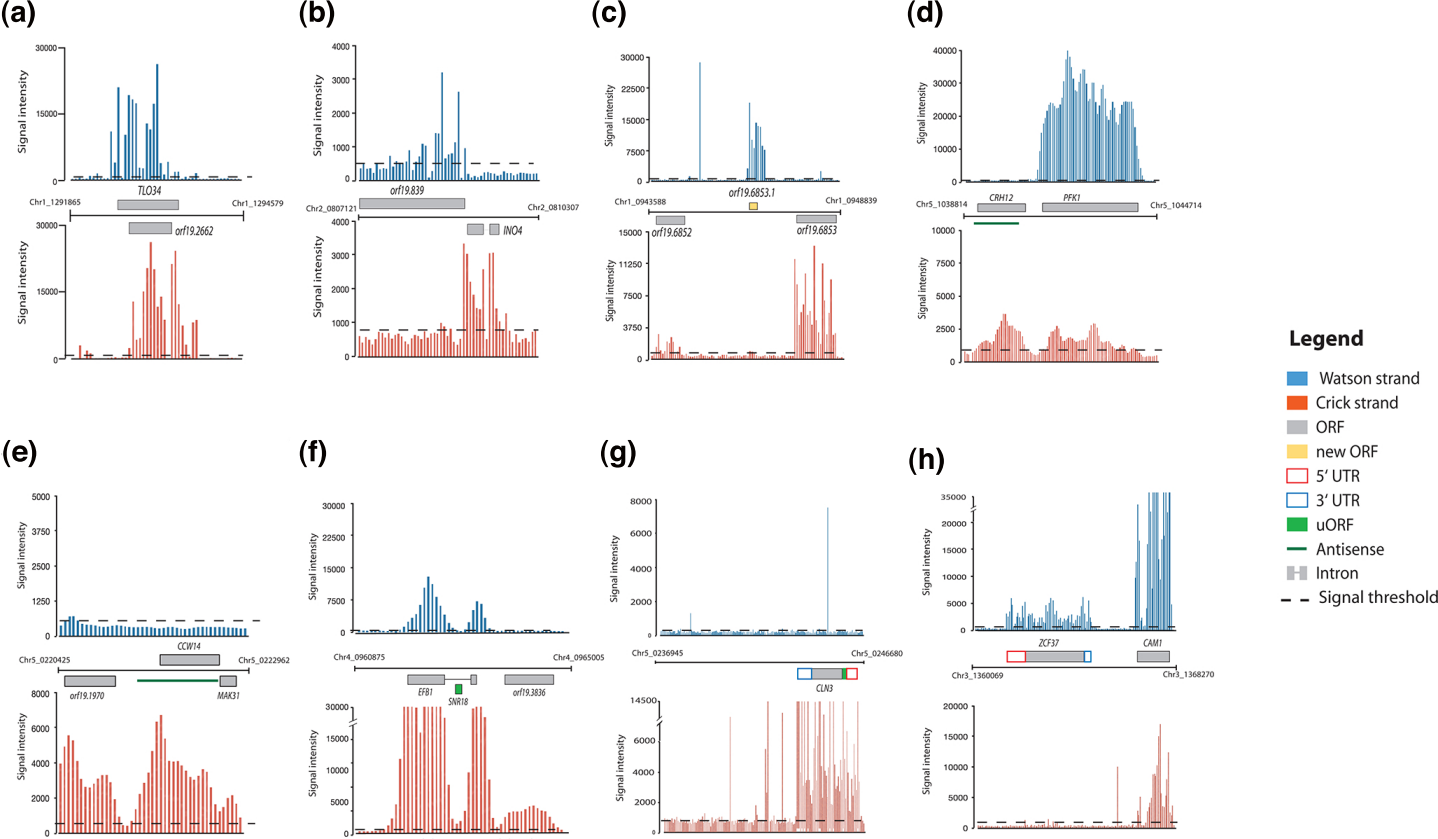
**General features of transcribed regions in the *C. albicans *genome**. Representative genes illustrating different transcriptional architectures are shown. **(a) **Nested genes. **(b) **Detection of *INO4 *intron. **(c) **Unannotated ORF. **(d, e) ***CRH12 *and *CCW14 *AS transcripts. **(f) **Intron-hosted snoRNA (*snR18*). **(g) **Putative conserved upstream ORF (uORF) of *CLN3*. **(h) **Unannotated 5' and 3' UTRs of *ZCF37*.

As shown in Figure 1, a clear correlation can be seen between the annotated ORFs and the signal intensities of probes. In general, the obtained data are in agreement with the current *Candida *Genome Database (CGD) annotation [27]. At the gene level, our data allowed us to confirm the presence of introns in a number of ORFs, as shown for *INO4 *(ORF19.837.1) and *EFB1 *(ORF19.3838) (Figure 2b, f). Although the resolution of our tiling array was not high enough to delimit precisely intron boundaries, we were able to confirm the introns previously annotated in the *C. albicans *genome [28]. Moreover, extensions of transcripts corresponding to potential upstream ORFs (for example, *CLN3*; Figure 2g) or 5' and 3' UTRs (for example, *ZCF37*; Figure 2h) were identified in several locations. Genetic elements displaying complex transcriptional architectures, such as nested genes (*TLO34 *and ORF9.2662; Figure 2a; Additional file 3) or intronic nested genes (*snR18 *hosted by the *EFB1 *intron; Figure 2f), were identified. Additionally, a large number of sense-AS transcript pairs have been detected (*PFK1 *and *EFB1*; Figure 2d, f). Intriguingly, in some cases, AS transcription was found on the opposite strand rather than the annotated strands (*CRH12 *and *CCW14*; Figure 2d, e). Previously unannotated ORFs and ncRNAs were also uncovered (ORF19.6853.1 and *snR18*; Figure 2c, f). To illustrate the annotation concept, some of the most relevant *C. albicans *genome features will be highlighted throughout the manuscript.

### Revisiting the *C. albicans *ORFeome

Based on the last CGD update (24 December 2009), the existing ORF catalogue of *C. albicans *consists of a total of 6,197 ORFs, of which 1,084 were experimentally verified, 4,933 functionally uncharacterized and 180 considered as dubious. In our current analysis, we have been able to detect the expression of 4,588 ORFs. Compared to other model organisms and despite the clinical relevance of the pathogenic yeast *C. albicans*, no comprehensive analysis has been done to provide experimental support to the *in silico*-based annotation. Our study thus provides such a genome-wide experimental validation for a large number of predicted ORFs for which transcription had not yet been confirmed by other approaches. Recently, using a comparative annotation approach, Butler *et al*. [12] identified 91 new ORFs, of which 80% are specific to the *Candida *clade. In the present study, 52% of those new ORFs (48 ORFs) were expressed above the background in our conditions, thus validating their functionality (Additional file 4). Furthermore, our data raised questions about 34 ORFs previously annotated as spurious or dubious [8] (Additional file 4). We also annotated 11 ORFs when screening the 2,172 expressed intergenic segments for their protein-coding potential (Additional file 4).

### Characterization of UTR regions

UTRs are known to play key roles in the post-transcriptional regulation of gene expression, influencing mRNA transport, mRNA subcellular localization, and RNA turnover [29]. Therefore, annotation of *C. albicans *UTRs has the potential to provide important insights into gene regulatory mechanisms underlying the biology and the pathogenicity of this fungus. To define *C. albicans *UTRs, we scanned the expression maps under different conditions and identified unannotated segments exhibiting an unbroken signal intensity connected to nuclear-encoded genes. A total of 481 5' UTRs and 846 3' UTRs longer than 240 bp were identified (Additional file 5). Compared to *Saccharomyces cerevisiae *and *Schizosaccharomyces pombe *[16,18,30], where the 3' UTRs are longer than 5' UTRs, the median length of both 5' and 3' UTRs was almost the same (the mean length of 5' and 3' UTRs was 88 bp and 84 bp, respectively, with a range of 0 to 3 kb for both 5' and 3' UTRs).

Genes with long 5' UTRs (>330 bp) were significantly enriched in regulatory functions, including transcription and signal transduction (Table 2; Additional file 6). A similar result was observed in *S. pombe *for both functions [31], and in *S. cerevisiae *for signal transduction [16]. In many eukaryotes, including the fission yeast *S. pombe*, it is well known that the most stable transcripts have short 5' UTRs, while the least stable transcripts have both long 5' and 3' UTRs [32,33].

**Table 2:** Gene Ontology analysis of genes with long 5' UTR regions (>330 bp)

GO terms	*P*-value	Median UTR length (bp)
DNA binding	2.36e-05	540
Transcription factor activity	3.68e-05	540
Phosphoprotein phosphatase activity	1.7e-04	420
Hyphal growth	2.60e-05	450
Filamentous growth	1.30e-10	480
Growth	1.54e-10	480
Regulation of biological process	4.70e-11	480
Cellular bud neck	3.6e-04	390

Intriguingly, a large number of transcripts with long 5' UTRs are key regulators of filamentous growth in *C. albicans*, including the transcription factors *EFG1*, *RFG1*, *CPH1*, *CPH2*, *CZF1*, *CRZ1*, *CRZ2*, *SSN6*, *NRG1 *and *FCR1*, and the phosphatases *YVH1*, *PTC8 *and *CPP1 *(Additional file 6). The regulation of RNA stability is a critical issue in modulating gene expression, in particular for transiently expressed regulatory genes such as those encoding transcription factors and phosphatases. Therefore, fine-tuning RNA turnover rates for those transcripts is potentially a key regulatory process involved in control of the yeast-to-hyphae transition in *C. albicans*. A high rate of RNA decay of transcripts involved in regulatory systems has been reported in *S. cerevisiae *as well [34]. Intriguingly, of the 38 RNAs identified recently as She3-transported in *C. albicans *during hyphal growth [35], 9 were found to exhibit long 5' UTRs (*P *= 4.3e-04). This leads us to speculate that long 5' UTRs are probably required for RNA transport to cellular locations where hyphal buds are produced.

### Widespread occurrence of antisense transcription in *C. albicans *

**Figure 3:**
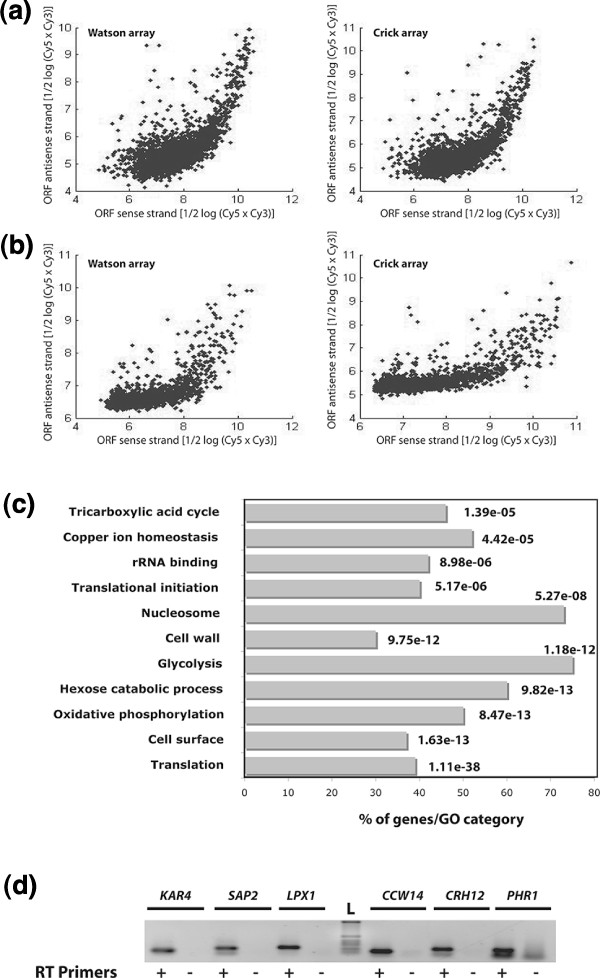
**Widespread occurrence of antisense transcription in *C. albicans***. **(a, b) **Scatter plots demonstrating the dependence of AS signal intensity on the sense expression level. Signal intensity of annotated feature (hyphae experiments) probes exhibiting an AS transcript expressed above the background were considered. The signals of probes representing either sense or AS transcripts for each hybridization performed without (a) or with (b) actinomycin D are plotted. **(c) **GO analysis of genes with recessive AS transcripts. The *P*-value was calculated using hypergeometric distribution, as described on the GO Term Finder website [27]. **(d) **Validation of dominant AS transcripts using strand-specific RT-PCR. RT-PCR analyses were performed on RNA from yeast cells using primers specific to the AS strand (**+**); samples were tested for endogenous RT priming and genomic DNA contamination (RT-PCR with no RT primers (**-**)).

Large-scale transcript mapping studies revealed the common occurrence of overlapping *cis*-natural AS transcripts in different model organisms [16-19,36]. In a recent study, Perocchi *et al*. [37] have shown that about half of all annotated antisense (AS) transcripts detected by tiling arrays in *S. cerevisiae *were experimental artifacts related to spurious synthesis of second-strand cDNAs that occurred during reverse transcription (RT) [37,38]. These authors showed that these RT artifacts were efficiently resolved by using the transcription inhibitor actinomycin D. In light of their finding, we have used actinomycin D to prevent the appearance of these artifacts. Indeed, as shown in Figure 3a, b, the use of actinomycin D reduced, in part, the dependence of AS signal intensity on the sense expression level.

AS transcription was observed for 724 genes, of which 623 are ORFs, 16 ncRNAs and 85 retrotransposons (Table S5 in Additional file 7). With few exceptions, all *C. albicans *AS transcripts belong to the completely overlapping natural AS transcript category. Based on sense/AS signal intensity ratio, AS transcripts were separated into two classes as was described for *S. cerevisiae *[37]. In the first class of AS transcripts, the hybridization signal intensity of the annotated features is higher and proportional to its AS counterpart (Figure 3a, b). This class contains the majority (79%) of the detected AS transcripts. Genes with this pattern are highly expressed in all conditions and GO analysis showed a preferential enrichment in housekeeping functions, including translation (*P *= 1.11e-38), cell surface proteins (*P *= 1.63e-13), glycolysis (*P *= 1.18e-12) and nucleosomes (*P *= 5.27e-08) (Figure 3c). Similar findings have been reported by experimental-based annotation of AS transcripts in wheat [39], rice [40] and *S. cerevisiae *[16], as well as by *in silico *approaches in other model organisms [41].

The second class of AS transcripts, where the average activity for the AS strand was much higher than the sense strand, contains only 37 genes (Figure 2d, e; Table S5 in Additional file 7). Strand-specific RT-PCR validated the expression of eight of these genes at the AS strand (Figure 3d). No functional enrichment was obtained for those transcripts. However, this AS category includes the transcription factor gene encoding the ortholog of *S. cerevisiae *Kar4p that plays a critical role in karyogamy during the mating process [42,43]. Overexpression of *KAR4 *in *S. cerevisiae *during vegetative growth causes a severe growth defect as a consequence of accumulation of cells arrested at G1 and G2/M stages [44]. Thus, if Kar4p plays a similar role in *C. albicans*, the AS transcription at this locus might be required for repression of the sense transcript during vegetative growth. A similar scenario was reported in *S. cerevisiae *where AS transcription opposite to *IME4 *has been shown to play a critical role in controlling entry into meiosis [45].

### RNAP-guided annotation of new *C. albicans *ncRNAs

Ongoing investigations on the function of ncRNAs established their specific roles in processes that require highly specific nucleic acid recognition without complex catalysis, such as guiding rRNA or tRNA covalent modifications [46,47] or guiding chromatin-modifying complexes to specific locations within the nucleus [48]. Given the central role of ncRNAs in such crucial biological processes, their genomic annotation is of great importance. However, annotating ncRNAs is a non-trivial task since their primary sequences are poorly conserved even between evolutionarily similar organisms. Here we adapted a strategy in which genome-wide occupancy of different subunits of RNAPs I, II and III is combined with expression data to annotate ncRNAs resulting from real transcriptional events. For this purpose we have performed chromatin immunoprecipitation on chip (ChIP-chip) of subunits that represent the three RNAP machines in *C. albicans *cells growing in rich media (YPD) at 30°C.

#### RNAP I-associated ncRNAs

RNAP I targets were determined by mapping the genomic location of the largest RNAP I subunit, Rpa190p (ORF19.1839). The results obtained show that Rpa190p occupancy was restricted to the rDNA locus where it binds the 18 S, the 5.8 S and the 28 S precursor gene promoters as well as internal transcribed regions (Additional file 8).

#### RNAP II-associated ncRNA

*In vivo *RNAP II occupancy was evaluated by performing ChIP-chip of the two subunits Rpo21p (ORF19.7655) and Rpb3p (ORF19.1248). Among the CGD-annotated ncRNAs, the snRNAs U1, U2, U4 and U5, associated with the spliceosomal machinery, were found to fit the established criteria. When Rpo21p and Rpb3 binding sites were matched to the 2,161 non-coding intergenic transfrags, 425 actively transcribed putative ncRNAs were found. A search of these 425 transfags using the *S. cerevisiae *ncRNA database returned only four matches that corresponded to snoRNAs. To generate an exhaustive list of *C. albicans *snoRNAs among the 2,161 ncRNA candidates, Snoscan [49] and snoGPS [50] servers were used to detect both C/D and H/ACA box snoRNA families, respectively. A total of 27 C/D box and 35 H/ACA box snoRNA candidates were identified. Most of the detected snoRNAs possess a canonical secondary structure and conserved C, D, A and ACA consensus motifs (Table S6 in Additional file 7). A comparison of these snoRNAs with entries in the Rfam database [51] returned 18 hits (4 H/ACA box and 14 C/D box) that match significantly to *S. cerevisiae *characterized snoRNAs. Orthologs of *S. cerevisiae *essential snoRNAs required for the cleavage of rRNA transcripts, namely U3a (snR17a), U3b (snR17b), U14 (snR128) and the snoRNA MRP *NME1*, were also detected and annotated in this study (Table S6 in Additional file 7). Interestingly, our results show that the U5 spliceosomal RNA (*SNRNAU5*) exhibits an extended transcriptional activity beyond its 3' terminal end, suggesting that *C. albicans*, like *S. cerevisiae*, possesses a long form of *SNRNAU5 *(U5L). Using 3' rapid amplification of cDNA ends (RACE), Mitrovich and Guthrie [52] have shown that, in addition to the vast majority of products that correspond to the short form of *SNRNAU5 *(U5S), a small amount of the long form was detected. In accordance with this, we found that the U5L transfrag was weakly transcribed compared to the U5 S. We also detected the previously characterized but unmapped *C. albicans *telomerase ncRNA *TER1 *[53] (Table S6 in Additional file 7). A total of 35 putative non-coding transfrags were randomly selected and their expression was confirmed using quantitative PCR (qPCR; Table S7 in Additional file 7). No obvious functions were attributed to the remaining 361 putative ncRNAs. Many large-scale gene expression mapping studies in mammals have suggested widespread transcription in intergenic regions that represent 47% to 80% of the transcribed features [54]. This 'dark matter' transcription has been accredited to previously undetected non-coding genes, 'junk' transcription, or experimental artifacts (reviewed in [15,55]). A recent report has demonstrated that the number and abundance of intergenic transcribed fragments from a large variety of different human and mouse tissue types were lower than shown earlier [54]. Using RNA-seq, van Bakel *et al*. [54] showed clearly that a significant number of these transcripts are associated with known genes and include many previously unidentified exons and alternative promoters. Though the majority of the 'dark matter' transcription seems to be artifactual, many conserved and presumably functional intergenic transcribed fragments remain to be characterized. In our work, many transfrags are conserved and expressed reproducibly in different conditions, suggesting a potential for a function and making them priority candidates for genetic perturbation and phenotypic characterization.

Additionally, to gain an insight into the function of these ncRNAs and their transcriptional regulation, we mapped the location of different transcription factors described in the literature for which genomic occupancies were determined using ChIP-chip. With the exception of Tbf1p, a master regulator of ribosomal protein expression in *C. albicans *[56,57], no transcription factors have been found associated with the promoter sequences of putative ncRNAs. Remarkably, in addition the occupancy of ribosomal protein genes and rRNA *cis*-regulatory regions, Tbf1p was found to be associated with the promoter of six snoRNAs annotated in this work. This finding implies that Tbf1p coordinates transcriptional activation of both structural components of the ribosome (rRNA and ribosomal protein genes) [56] in addition to the snoRNAs that guide methylation and pseudouridylation modifications required for ribosome maturation and functionality. Recently, Preti *et al*. [58] showed that Tbf1p in *S. cerevisiae *is required for the activation of snoRNA, implying a similar role in *C. albicans*. Similar findings were also obtained in the plant model *Arabidopsis thaliana *where the Tbf1p motif (ACCCTA) was significantly enriched in upstream snoRNAs (*P *= 4.64e-20), suggesting a highly conserved role for this factor.

#### RNAP III-associated ncRNAs

In eukaryotic cells, RNAP III transcribes genes encoding tRNAs, 5 S rRNA and other ncRNAs, such as the RNA component of RNase P (*RPR1*) and the U6 snRNA (*SNR6*) [59-61]. To investigate the targets of the RNAP III machinery in *C. albicans*, we performed ChIP-chip with the subunit Rpc82p (ORF19.2847). Based simply on signal intensities of the ChIP-chip, Rpc82p targets can be divided in two categories. The first category includes loci with a high level of occupancy (between 6- and 45-fold enrichment): this category contains 120 tRNAs and the 5 S rRNA (Table S8 in Additional file 9) alongside the well-known non-tRNA genes transcribed by RNAP III (*RPR1*, *SNR6*, *snR52*, *SCR1*), which were characterized [62,63] but not mapped (Additional file 10). For all these binding events significant transcriptional hybridization signals were detected at least in two different conditions for 67 tRNAs, *RPR1*, *SNR6*, *snR52*, *SCR1 *and the 5 S rRNA. The second category includes loci with a low level of occupancy (between 2- and 4.5-fold enrichment): with a few exceptions, all these loci were expressed and correspond to repetitive DNA elements associated with retrotransposons. Since long terminal repeat (LTR) retrotransposons are present in the *C. albicans *genome in multiple copies and often adjacent to tRNAs, the occupancy of Rcp82p at these loci is most probably a result of an amplification of cross-hybridization signals.

It has been demonstrated that the yeast *S. cerevisiae *LTR retrotransposons Ty1 and Ty3 strictly target regions in the vicinity of tRNAs [64,65]. This conserved strategy is most likely adopted to avoid deleterious integrations into coding sequences. In the social amoeba *Dictyostelium discoideum*, Siol *et al*. [66] have demonstrated that the general transcription factor TFIIIC of the RNAP III machinery is actively required for targeted integration of the retrotransposon TRE5-A [66]. This finding supports that, in our study, some Rpc82p-retrotransposon-occupied loci might be real binding events. Indeed, based on binding intensity, it is probably the case for two loci where Rpc82p was found to bind the repetitive DNA elements beta-1a and beta-1c of the retrotransposon Tca8 with an occupancy level similar to that seen for tRNAs (Table S8 in Additional file 9).

### Subtelomeric regions are transcriptionally active and express a cluster of ncRNAs

**Figure 4:**
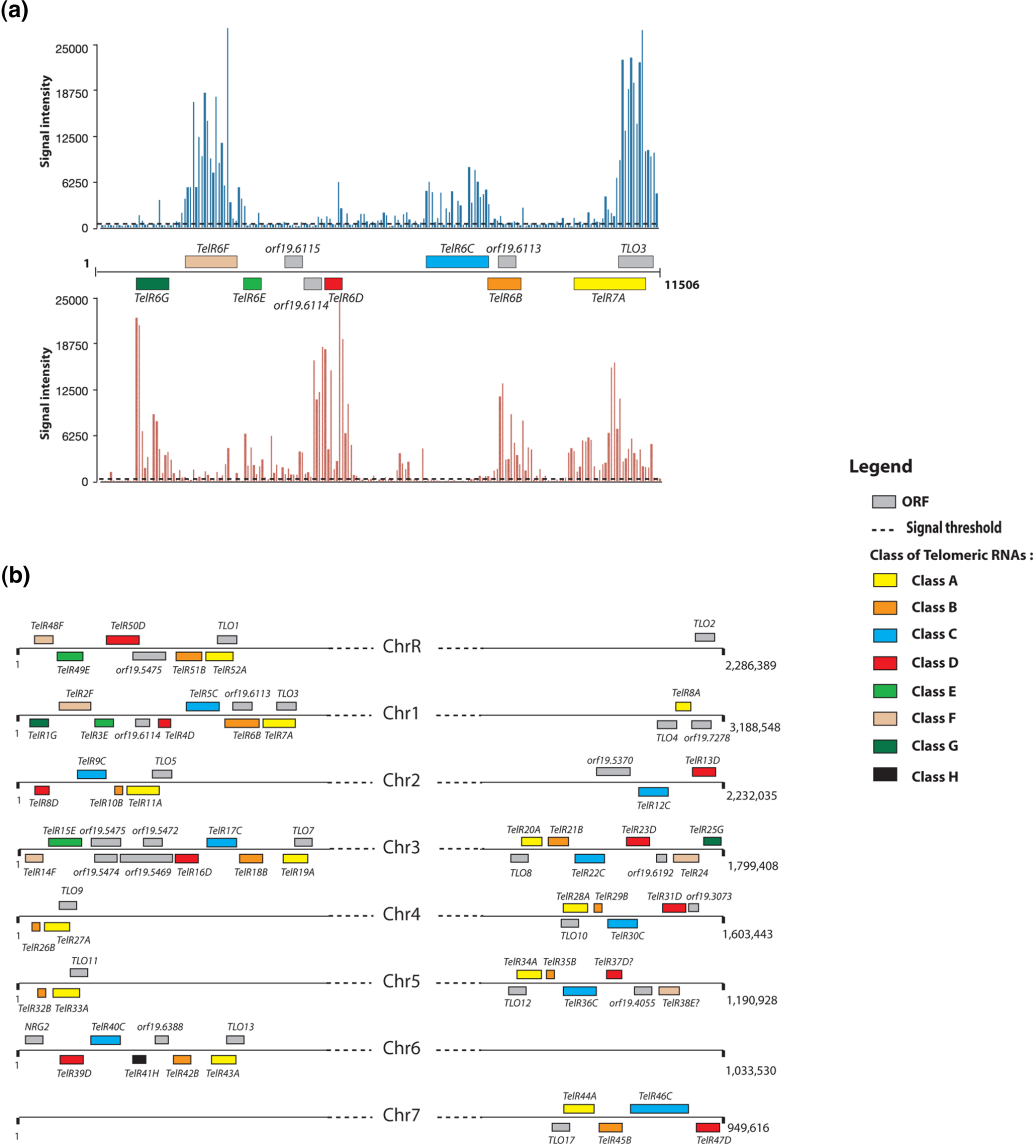
**Subtelomeric regions bear transcriptionally active clusters of ncRNAs**. **(a) **Genomic overview of subtelomeric regions of the left arm of chromosome 1 showing a cluster of transcribed segments with no protein-coding potential. Different classes of TelRs are represented. **(b) **Schematic representation of genomic organization of the different classes of TelRs at chromosome arms. *TLO *genes along with subtelomeric ORFs are shown.

We found that clustered transcribed segments (52 transfrags) with no protein-coding potential were located at the subtelomeric regions of all chromosomes (Figure 4a). This finding is in accordance with early work in mammals that established that telomeres, originally thought to be transcriptionally silent, bore actively transcribed ncRNAs [67,68]. Based on sequence similarity, these telomere-associated ncRNAs (TelRs) can be divided into eight classes (TelR A to H; Figure 4; Table S9 in Additional file 9). With no exception, all TelRs from class A are AS of *TLO *genes, overlapping with their 5' ends. The class B TelRs correspond to the telomeric element CARE-2 [69], which is composed, in part, of the LTR retrotransposon. TelRs are specific to *C. albicans *and their sequences are not conserved throughout the clades represented in the CTG. Furthermore, when TelR sequences of the SC5314 strain were compared to their counterparts in the WO1 strain, we noticed a significant degree of polymorphism. Subtelomeric regions are suggested to be potential locations of gene amplification since one telomere might be functionally exchanged with another [70]. Thus, in addition to *TLO *genes, TelRNAs seem to be members of a new family of multi-copy subtelomeric ncRNAs.

### Differentially regulated transfrags during pathogenic-related growth

**Figure 5:**
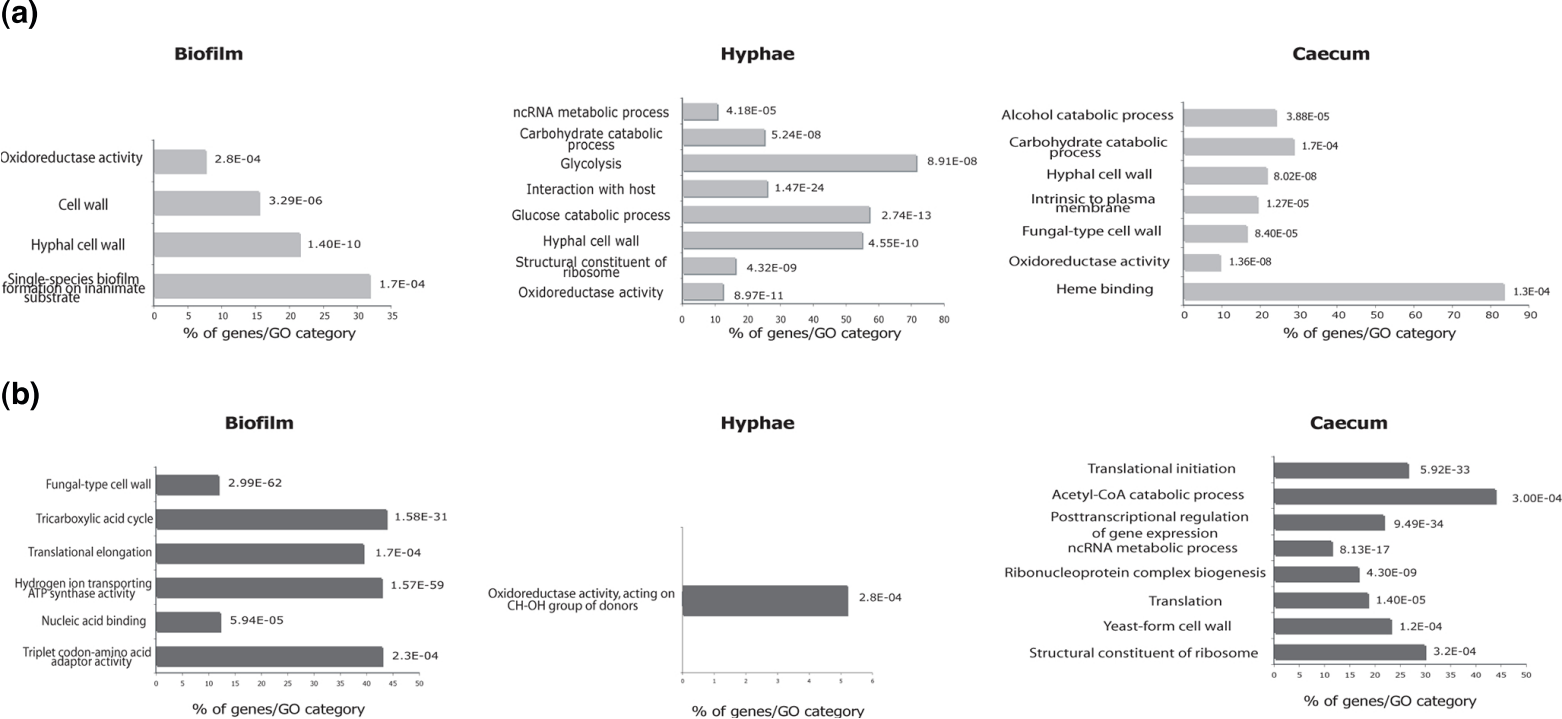
**Functional gene categories differentially regulated in hyphae, biofilm and caecum-grown cells**. GO functional categories of **(a) **up- and **(b) **down-regulated genes are shown. *P*-values were calculated using hypergeometric distribution.

As an opportunistic fungus, *C. albicans *must activate numerous transcriptional outputs to promote host colonization or virulence [71]. To elucidate the transcriptional patterns of annotated features in the different tested conditions, signal intensities of transfrags detected in cells growing as hyphae, biofilms and in the mouse caecum were compared to their counterparts in yeast cells (the control condition). GO analysis was used to assess the average expression levels of genes encoding specific classes of proteins in the three tested conditions (Figure 5; Additional files 11 and 12). In general, our results demonstrated a large overlap in transcripts present in hyphae or biofilms that were found in other studies. For instance, many differentially expressed genes in the three tested conditions encode adhesins and fungal cell wall proteins, consistent with their described roles during the interaction with the host and biofilm formation [71-73]. Unexpectedly, classes of genes involved in ncRNA metabolic processes, such as small nucleolar ribonucleoprotein (snoRNP) assembly complexes, were found differentially expressed in hyphae and in cells recovered from the caecum (Figure 5). Similarly, several genes that had never been detected before in *C. albicans *biofilms, including genes encoding tRNAs (GO term 'translation elongation'; *P *= 1.57e-59), were found to be significantly consistently repressed with the repression of ribosomal genes, as reported in other biofilm models [74,75].

Interestingly, we found that genes encoding proteins involved in heme binding were actively transcribed in *C. albicans *cells recovered from the caecum (Figure 5a), suggesting that the caecum is an iron-poor niche. These genes include hemoglobin-receptors *RBT5*, *PGA10*, *CSA1*, and *DAP1*, as well as the heme-degradation oxygenase *HMX1*. During this commensal growth, *C. albicans *also activates genes related to carbohydrate catabolism, as was reported in other *in vivo *infection models [71]. qPCR confirmed the activation of selected genes representing carbohydrate catabolism and heme binding functions in two independent biological replicates (Additional file 13).

To discover candidate ncRNAs potentially associated with host-dependant growth, we defined differentially expressed intergenic transfrags in *C. albicans *cells growing in the caecum as well as in cells undergoing hyphal and biofilm growth. Using a stringent cutoff (see Materials and methods), 264, 47, and 64 transfrags were found differentially regulated in caecum-grown cells, hyphae and biofilm cells, respectively (Additional file 14). Many of them are bound by the RNAP II or are conserved with other species from the *Candida *clade (Additional file 14), suggesting a significant potential for function.

## Conclusions

We provide a comprehensive expression map that covers a set of conditions relevant to *C. albicans *pathogenic developmental stages. The identification of unannotated transcribed regions was the main motivation of this study. Using multiple genome-scale measurements (expression profiling and RNAP occupancy), we have characterized and annotated a number of ncRNAs hidden in the 'dark matter' of the *C. albicans *genome. These ncRNAs candidates constitute an interesting framework for future functional studies and will contribute to our understanding of the role of the *C. albicans *non-coding genome. Furthermore, our work has uncovered different genetic features, including extensive AS transcription, 5' and 3' UTRs and expression at subtelomeric regions. One particular feature was the enrichment of genes with long 5' UTRs in regulatory function associated with hyphal development. This feature might imply noteworthy regulation at the post-transcriptional level of the *C. albicans *yeast-to-hyphae switch and should be clarified in the near future. Transcript mapping data and RNAP occupancies will be available at the CGD database [76] displayed via a genome browser interface (Gbrowse), enabling the inspection of any locus of interest.

## Materials and methods

### Growth media and conditions

Strains used in this study are listed in Additional file 15. For general propagation and maintenance conditions, the strains were cultured at 30°C in yeast-peptone-dextrose (YPD) medium supplemented with uridine (2% Bacto peptone, 1% yeast extract, 2% dextrose, and 50 μg/ml uridine, with the addition of 2% agar for solid medium). Cell growth, transformation and DNA preparation were carried out using standard yeast procedures.

For gene expression profiling of yeast-form cells, saturated overnight cultures of the SC5314 strain were diluted to a starting OD_600 _of 0.1 in 50 ml fresh YPD and grown at 30°C to an OD_600 _of 0.8. Hyphae were induced by growing *Candida *cells in YPD plus 10% fetal bovine serum at 37°C to an OD_600 _of 0.8. Cultures were harvested by centrifugation at 3,000 × g for 5 minutes, and the pellet rapidly frozen in liquid nitrogen. Biofilms were grown in RPMI medium at 37°C as described [77]. For RNA extracted from caecum-grown cells, female C57BL/6 mice (5 to 7 weeks old) were treated with tetracycline (1 mg/ml), streptomycin (2 mg/ml) and gentamicin (0.1 mg/ml) added to their drinking water for the duration of the experiment, beginning 4 days prior to inoculation. *C. albicans *cells (5 × 10^7 ^cells) were orally inoculated into the mice by gavage. Three days post-inoculation, the mice were sacrificed and the contents of the caecum were recovered and frozen in RNALater (Ambion, Austin, TX, USA) at -80°C. Caecum contents were filtered through 500 μm polypropylene mesh (Small Parts, Inc., Miramar, FL, USA) to remove large particles and RNA was extracted by bead beating with 0.5 mm zirconia/silica beads in TRIzol (Invitrogen, Carlsbad, CA, USA). After the TRIzol RNA purification procedure described by the manufacturer, RNA was further purified on Qiagen (Valencia, CA, USA) columns with on-column DNase treatment.

### Tiling array design

Starting from sequences from the *C. albicans *Genome Assembly 21 [9] and the *MTL *alpha locus [78], we extracted a continuous series of 242,860 60-bp oligonucleotides each overlapping by 1 bp. We then eliminated 2,062 probes containing stretches of 13 or more A or T nucleotides. The remaining 240,798 sequences were then used to produce sense and AS whole genome tiling arrays using the Agilent Technologies eArray service.

### Microarray experiments

To extract RNA from cells, samples stored at -80°C were placed on ice and RNeasy buffer RLT was added to pellets at a ratio of 10:1 (vol/vol) buffer/pellet. The pellet was allowed to thaw in the buffer with vortexing briefly at high speed. The resuspended pellet was placed back on ice and divided into 1 ml aliquots in 2 ml screw cap microcentrifuge tubes containing 0.6 ml of 3 mm diameter acid-washed glass beads. Samples were homogenized 5 times, 1 minute each, at 4,200 RPM using Beadbeater. Samples were placed on ice for 1 minute after each homogenization step. After the homogenization the Qiagen RNeasy protocol was followed as recommended. Total RNA samples were eluted in RNAse free H_2_O. RNA quality and integrity were assessed using an Agilent 2100 bioanalyzer.

cDNA labeling and microarray production were performed as described [79]. Briefly, 20 μg of total RNA was reverse transcribed using 9 ng of oligo(dT)_21 _and 15 ng of random octamers (Invitrogen) in the presence of Cy3 or Cy5-dCTP (Invitrogen) and 400 U of Superscript III reverse transcriptase (Invitrogen). Actinomycin D was used to inhibit synthesis of the second cDNA strand to a final concentration of 6 μg/ml.

To assess actinomycin D efficiency in resolving spurious AS transcripts, signal intensities of annotated feature (from yeast and hyphae experiments) probes exhibiting an AS transcript expressed above the background were considered. The signals of every probe representing either sense or AS transcripts for each hybridization, performed with or without actinomycin D, were plotted (Figure 3a, b).

After cDNA synthesis, template RNA was degraded by adding 2.5 units RNase H (Promega, Madison, WI, USA) and 1 μg RNase A (Pharmacia, Uppsala, Sweden) followed by incubation for 15 minutes at 37°C. The labeled cDNAs were purified with a QIAquick PCR Purification Kit (Qiagen). Prior to hybridization, Cy3/Cy5-labeled cDNA was quantified using a ND-1000 UV-VIS spectrophotometer (NanoDrop, Wilmington, DE, USA) to confirm dye incorporation. DNA microarrays were processed and analyzed as previously described [80].

### Whole-genome location profiling by ChIP-chip and data analysis

*RPA190 *(ORF19.1839), *RPC82 *(ORF9.2847), *RPB3 *(ORF19.1248) and *RPO21 *(ORF19.7655) were TAP-tagged *in vivo *with a TAP-*URA3 *PCR product as described [81]. Transformants were selected on YPD -ura plates and correct integration of the TAP-tag was checked by PCR and sequencing. Cells were grown to an OD_600 nm _of 2 in 40 ml of YPD. The subsequent steps of DNA cross-linking, DNA shearing, chromatin immuno-precipitation and DNA labeling with Cy dyes were conducted exactly as described by Lavoie *et al*. [81]. Tiling arrays were co-hybridized with tagged immunoprecipitated (Cy5-labeled) and mock immunoprecipitated (untagged BWP17 strain; Cy3-labeled) DNA samples. Microarray hybridization, washing and scanning were performed as described above. The significance cut-off was determined using the distribution of log-ratios for each factor. It was set at 2 standard deviations from the mean of log-transformed fold enrichments. Values shown are an average of two biological replicates derived from independently isolated transformants of tagged and mock constructs. Peak detection was performed using Gaussian edge detection applied to the smoothed probe signal curve as described [82].

### Expression analysis by real-time quantitative PCR

For qPCR, cDNA was synthesized from 5 μg of total RNA using the RT system (50 mM Tris-HCl, 75 mM KCl, 5 mM dithiothreitol, 3 mM MgCl_2_, 400 nM oligo(dT)_15_, 20 ng random octamers, 0.5 mM dNTPs, 200 units Superscript III reverse transcriptase; Invitrogen). The mixture was incubated for 60 minutes at 50°C. cDNAs were then treated with 2 U of RNase H (Promega) for 20 minutes at 37°C followed by heat inactivation of the enzyme at 80°C for 10 minutes. Aliquots were used for qPCR, which was performed using the Mx3000P QPCR System (Agilent, Santa Clara, CA, USA) with the QuantiTect SYBR Green PCR master mix (Qiagen). Cycling was 10 minutes at 95°C followed by 40 cycles (95°C, 10 s; 58°C, 15 s; 72°C, 15 s). Samples were done in triplicate and means were used for calculations. Fold changes were estimated using the coding sequence of the *C. albicans ACT1 *ORF as a reference. Fold enrichments of the tested coding sequences were estimated using the comparative ΔΔCt method as described [83]. Primers used for qPCR are summarized in Additional file 16.

### Strand-specific RT-PCR

Strand-specific RT was performed as for the qPCR experiment. The RT reaction used 2 pmol of gene-specific primers (Additional file 16) designed to anneal to the AS transcript. Strand-specific RT-PCR was performed using 1 μl of the RT reaction. Cycling was 10 minutes at 95°C followed by 30 cycles (95°C, 10 s; 60°C, 55 s; 72°C, 30 s). As a negative control, RT-PCR was performed using RT reactions in which reverse transcriptase was not added.

### Genome annotation and DNA sequence conservation

The DNA sequence and annotation of *C. albicans *assembly 21 were obtained from CGD [27]. The genome of the closely related species *C. dubliniensis *was obtained from the Sanger Institute [84]. Conserved regions of *C. albicans *were defined as regions where significant alignments (e-value <1e-10) were found with *C. dubliniensis *using the blast program [85].

### Threshold levels, transfrags and peak detection

A background value was established for every channel of all transcription mapping on the tiling arrays based on the 95th percentile of the distribution of the median expression level of unannotated non-conserved regions of the genomes. In all, 3,178 regions spanning at least 3 probes (>180 bp) were used to establish this stringent detection threshold. Furthermore, an annotated feature (ORF, RNA or retrotransposons) was considered expressed only if the mean expression levels of both the Cy3 and Cy5 channels were above their respective threshold levels.

Before the detection of unannotated intergenic transcribed regions, a median filter (n = 3) was applied to the tiling data to eliminate single isolated probes with excessively high values. A Gaussian smoothing function was then applied and regions that spanned consecutive probes above the background were reported. Based on the presence and expression level of adjacent annotated features, these transfrags were classified as UTR or intergenic. A transfrag was considered as an ORF if it is longer than 50 codons. Differential expression levels of each probe were taken as the log2 of theratio (Cy3/Cy5) normalized using locally weighted scatter plot smoothing(LOWESS). Annotated or newly discovered intergenic regions differentially expressed were taken as the mean value of the probes covering these regions. Peak location and detection were performed as exactly described by Lavoie *et al*. [57].

GO annotation was performed using the GO Term Finder at the CGD website [27]. The *P*-value was calculated using hypergeometric distribution, as described on the GO Term Finder website. Motif detection of *A. thaliana *snoRNA promoters was performed using the TAIR Motif Finder tool [86].

### Accession codes

Microarray data have been submitted to the NCBI Gene Expression Omnibus (GEO) under accession number [GEO:GSE22625].

## Abbreviations

AS: antisense; bp: base pair; CGD: *Candida *Genome Database; ChIP-chip: chromatin immunoprecipitation on chip; GO: Gene Ontology; LTR: long terminal repeat; ncRNA: non-coding RNA; ORF: open reading frame; qPCR: quantitative PCR; RNAP: RNA polymerase; RT: reverse transcription; snRNA: small nuclear RNA; snoRNA: small nucleolar RNA; TelR: telomere-associated ncRNA; UTR: untranslated region.

## Authors' contributions

AS and AN conceived and designed the experiments. AS performed the experiments with the help of CA and FT. AS and HH analyzed the data. CK, MvhH and HL contributed regents, materials and analysis tools. AS wrote the paper. AN and MW reviewed and edited the paper. All authors read and approved the final manuscript.

## Supplementary Material

Additional file 1 **Figure S1**. GO analysis of the 28% of nuclear genes not expressed in this study. Click here for file

Additional file 2 **Table S1**. Genome-scale detection of unannotated transcribed segments in *C. albicans *growing in different conditions. Click here for file

Additional file 3 **Table S15**. List of nested or overlapping genes validated in this work. Click here for file

Additional file 4 **Table S2**. List of detected ORFs and pseudogenes. Click here for file

Additional file 5 **Table S3**. List of ORFs exhibiting long 5' and 3' UTRs (>240 bp) Click here for file

Additional file 6 **Table S4**. Gene Ontology analysis of ORFs with long 5' and 3' UTR regions (>330 bp). Click here for file

Additional file 7 **Tables S5, S6, and S7**. Genome-wide detection of ncRNAs: Table S5, AS transcripts; Table S6, housekeeping ncRNAs; and Table S7, RT-qPCR validation of randomly selected ncRNAs. Click here for file

Additional file 8 **Figure S2**. Transcription and RNAP I and III occupancies within the rDNA locus. Click here for file

Additional file 9 **Tables S8 and S9**. Detection of RNAP III binding peaks (Table S8) and genomic organization and coordinates of telomeric ncRNA (TelRs; Table S9). Click here for file

Additional file 10 **Figure S3**. Transcription and RNAP III occupancy of ncRNAs. tRNAs (a, b), *RPR1 *(b) and an unknown ncRNA (c) are represented. Click here for file

Additional file 11 **Table S10**. GO process annotation of differentially regulated annotated features using the CGD GO Term Finder [27]. Click here for file

Additional file 12 **Table S11**. List of differentially expressed ORFs in hyphae, biofilm and caecum-grown cells. Click here for file

Additional file 13 **Figure S4**. Real-time quantitative PCR validation of candidate genes differentially expressed in caecum-grown *Candida *cells. Both heme-binding (a) and carbohydrate catabolism genes (b) were considered. Click here for file

Additional file 14 **Table S12**. Genome-scale detection of differentially expressed unannotated transfrags in *C. albicans*. Click here for file

Additional file 15 **Table S13**. *C*. *albicans *strains used in the study [87,88]. Click here for file

Additional file 16 **Table S14**. Primers used in this study. Click here for file
