# Supplementary figures and images for: Experimental annotation of the human pathogen Candida albicans coding and noncoding transcribed regions using high-resolution tiling arrays
Source: Genome Biol. 2010 Jul 9;11(7):R71. doi: 10.1186/gb-2010-11-7-r71 (PMC2926782; doi:10.1186/gb-2010-11-7-r71)

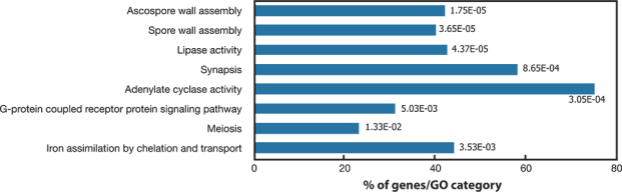

Supplement: Additional file 1 — Figure S1. GO analysis of the 28% of nuclear genes not expressed in this study. [file gb-2010-11-7-r71-S1.pdf]

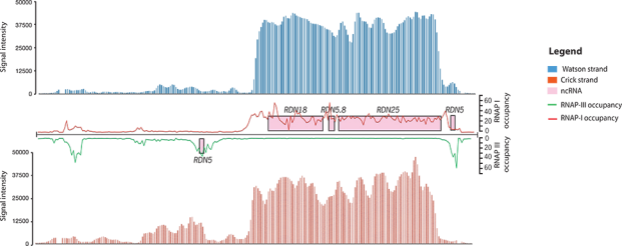

Supplement: Additional file 8 — Figure S2. Transcription and RNAP I and III occupancies within the rDNA locus. [file gb-2010-11-7-r71-S8.pdf]

**(a)****Replicate 1**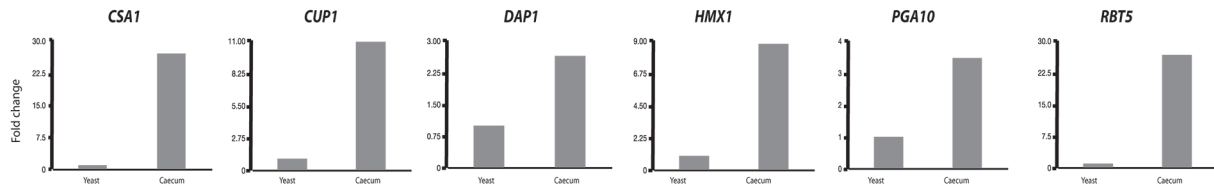**Replicate 2**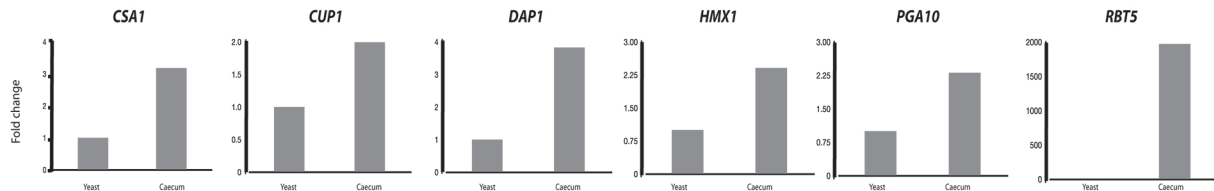**(b)****Replicate 1**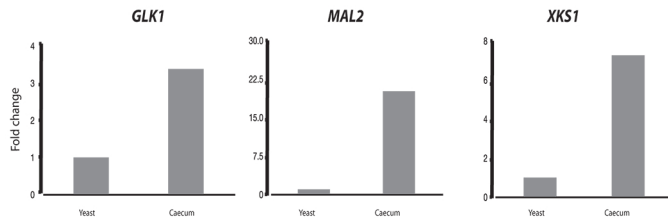**Replicate 2**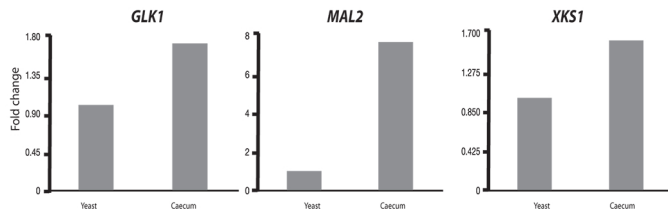

Supplement: Additional file 13 — Figure S4. Real-time quantitative PCR validation of candidate genes differentially expressed in caecum-grown Candida cells. Both heme-binding (a) and carbohydrate catabolism genes (b) were considered. [file gb-2010-11-7-r71-S13.pdf]
