# Supplementary material for: Experimental annotation of the human pathogen Candida albicans coding and noncoding transcribed regions using high-resolution tiling arrays
Source: Genome Biol. 2010 Jul 9;11(7):R71. doi: 10.1186/gb-2010-11-7-r71 (PMC2926782; doi:10.1186/gb-2010-11-7-r71)
Supplement: Additional file 6 — Table S4. Gene Ontology analysis of ORFs with long 5' and 3' UTR regions (>330 bp). [file gb-2010-11-7-r71-S6.doc]

| **GO terms** | **Genes** | ***P*-Value** |
| --- | --- | --- |
| **5’ UTR** | | |
| **GO Biological Process** | | |
| **Regulation of biological process** | *AAF1, ADH1, BMH1, CAS5, CDC20, CDC39, CLA4, CLB4, CLN3, CPH1, CPH2, CPP1, CRZ1, CRZ2, CSC25, CTA24, CZF1, ECM22, EFG1, FCR1, FUN31, GAC1, GCN4, HHT2, IRA2, MPT5, MSB2, MSS4, NHP6A, NRG1, PIN4, PTP3, REG1, RFG1, RGA2, RTF1, RTS1, SET3, SFL2, SFU1, SMI1, SOK1, SSN6, SSU81, TAR1, UPC2, WHI3, YVH1, orf19.1619, orf19.1769, orf19.2631, orf19.3302, orf19.3751, orf19.4347, orf19.4437, orf19.4488, orf19.4502, orf19.4657, orf19.5026, orf19.5160, orf19.7368, orf19.7489* | 4.70e-11 |
| **Growth** | *AAF1, ALS2, BMH1, BNI4, CAS5, CDC39, CDC4, CLA4, CLB4, CLN3, CPH1, CPH2, CPP1, CRZ1, CSC25, CZF1, EFG1, ERG3, FCR1, GCN4, KSP1, MSB2, MYO2, NRG1, PIN4, PTC8, RFG1, RGA2, SEP7, SET3, SFL2, SPT3, SSN6, SSU81, VAC8, WHI3, YVH1, ZCF37, orf19.1536, orf19.2892, orf19.5406, orf19.6705, orf19.921* | 1.54e-10 |
| **Filamentous growth** | *AAF1, ALS2, BMH1, BNI4, CAS5, CDC39, CDC4, CLA4, CLB4, CLN3, CPH1, CPH2, CPP1, CRZ1, CSC25, CZF1, EFG1, ERG3, FCR1, GCN4, KSP1, MSB2, MYO2, NRG1, PIN4, PTC8, RFG1, RGA2, SEP7, SET3, SFL2, SPT3, SSN6, SSU81, VAC8, WHI3, YVH1, ZCF37, orf19.1536, orf19.5406, orf19.6705, orf19.921* | 1.30e-10 |
| **Regulation of cellular metabolic process** | *AAF1, BMH1, CAS5, CDC20, CDC39, CLB4, CLN3, CPH1, CPH2, CRZ1, CRZ2, CTA24, CZF1, ECM22, EFG1, FCR1, FUN31, GAC1, GCN4, IRA2, MPT5, NHP6A, NRG1, PTP3, REG1, RFG1, SET3, SFL2, SFU1, SSN6, TAR1, UPC2, orf19.1619, orf19.1769, orf19.2631, orf19.3302, orf19.4347, orf19.4437, orf19.4488, orf19.4502, orf19.5026, orf19.7368* | 7.08e-07 |
| **Regulation of transcription, DNA-dependent** | *AAF1, CAS5, CDC39, CLN3, CPH1, CPH2, CRZ1, CRZ2, CTA24, CZF1, ECM22, EFG1, FCR1, GAC1, GCN4, MPT5, NHP6A, NRG1, REG1, RFG1, SFL2, SFU1, SSN6, UPC2, orf19.1619, orf19.1769, orf19.2631, orf19.3302, orf19.4347, orf19.4437, orf19.4488, orf19.4502, orf19.5026* | 1.02e-05 |
| **Hyphal growth** | *ALS2, CAS5, CLA4, CLN3, CPH1, CPH2, CPP1, CRZ1, EFG1, ERG3, GCN4, MYO2, NRG1, PTC8, RFG1, RGA2, SEP7, SSN6, VAC8, YVH1* | 2.60e-05 |
| **Response to pheromone** | *ALS3, ALS5, CDC39, CLA4, CPH1, CPP1, MPT5, PTP3, RGA2, orf19.1769, orf19.4347* | 8.5e-04 |
| **GO Molecular function** | | |
| **DNA binding** | *BMH1, CAS5, CPH1, CPH2, CRZ1, CRZ2, CZF1, ECM22, EFG1, FCR1, FIL2, GCN4, HHT2, NHP6A, NRG1, RFG1, RME1, SFU1, UPC2, ZCF37, orf19.1052, orf19.1189, orf19.3428, orf19.4123, orf19.4437, orf19.4502, orf19.5026, orf19.5953* | 2.36e-05 |
| **Transcription factor activity** | *CAS5, CPH1, CPH2, CRZ1, CRZ2, CZF1, ECM22, EFG1, FCR1, FIL2, GCN4, NRG1, RFG1, RME1, SFU1, UPC2, ZCF37, orf19.5026, orf19.5953* | 3.68e-05 |
| **Phosphoprotein phosphatase activity** | *CPP1, PTC4, PTC8, PTP3, RTS1, YVH1, orf19.4657, orf19.5160, orf19.5406* | 1.7e-04 |
| **GO Cellular component** | | |
| **Cellular bud neck** | *CHS4, MYO2, RGA2, RTS1, SEP7, SMI1, orf19.3751, orf19.6705, orf19.7489* | 3.6e-04 |
| **3’UTR** | | |
| **GO Cellular component** | | |
| **Fungal-type cell wall** | *ALS2, BMH1, EAP1, EFT2, IPP1, MET6, PGA59, PGA6, PGA62, PIR1, SSR1, SUR7, TOS1, TSA1, WSC1, XOG1, YWP1* | 5e-04 |

**Table S4. Gene ontology analysis of ORFs with long 5’- and 3’-UTR regions (> 330bp).**
