# Supplementary material for: Experimental annotation of the human pathogen Candida albicans coding and noncoding transcribed regions using high-resolution tiling arrays
Source: Genome Biol. 2010 Jul 9;11(7):R71. doi: 10.1186/gb-2010-11-7-r71 (PMC2926782; doi:10.1186/gb-2010-11-7-r71)
Supplement: Additional file 10 — Figure S3. Transcription and RNAP III occupancy of ncRNAs. tRNAs (a, b), RPR1 (b) and an unknown ncRNA (c) are represented. [file gb-2010-11-7-r71-S10.pdf]

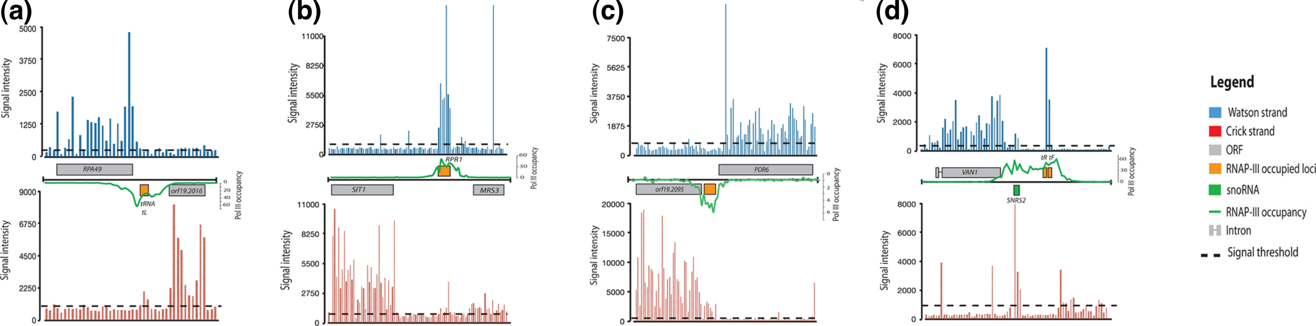

(a)
(b)
(c)
(d)
Signal intensity
RPA49
tRNA
tL
orf19.2016
Pol III occupancy
RPR1
SIT1
MRS3
PDR6
orf19.2095
VAN1
tR tF
SNR52
Legend
Watson strand
Crick strand
ORF
RNAP-III occupied loci
snoRNA
RNAP-III occupancy
Intron
Signal threshold
