# Supplementary material for: Experimental annotation of the human pathogen Candida albicans coding and noncoding transcribed regions using high-resolution tiling arrays
Source: Genome Biol. 2010 Jul 9;11(7):R71. doi: 10.1186/gb-2010-11-7-r71 (PMC2926782; doi:10.1186/gb-2010-11-7-r71)
Supplement: Additional file 15 — Table S13. C. albicans strains used in the study [87,88]. [file gb-2010-11-7-r71-S15.doc]

| Strain | Genotype | Source |
| --- | --- | --- |
| SC5314 | *Wild type (clinical strain)* | [87] |
| BWP17 | *his1/his1 ura3/ura3 arg4/arg4* | [88] |
| AS50 | *his1/his1 arg4/arg4::RPA190-TAP-URA3/ura3* | This study |
| AS51 | *his1/his1 arg4/arg4::RPB3-TAP-URA3/ura3* | This study |
| AS52 | *his1/his1 arg4/arg4::RPO21-TAP-URA3/ura3* | This study |
| AS53 | *his1/his1 arg4/arg4::RPC82-TAP-URA3/ura3* | This study |

Table S13. *Candida* *albicans* strains used in the study
